# Fear influences phantom sound percepts in an anechoic room

**DOI:** 10.3389/fpsyg.2022.974718

**Published:** 2022-09-26

**Authors:** Sam Denys, Rilana F. F. Cima, Thomas E. Fuller, An-Sofie Ceresa, Lauren Blockmans, Johan W. S. Vlaeyen, Nicolas Verhaert

**Affiliations:** ^1^Research Group Experimental Otorhinolaryngology (ExpORL), Department of Neurosciences, University of Leuven, Leuven, Belgium; ^2^Department of Otorhinolaryngology–Head and Neck Surgery, University Hospitals of Leuven, Leuven, Belgium; ^3^Multidisciplinary University Center for Speech-Language Pathology and Audiology, University Hospitals of Leuven, Leuven, Belgium; ^4^Research Group Health Psychology, Department of Behavior, Health and Psychopathology, University of Leuven, Leuven, Belgium; ^5^Department of Clinical Psychological Science, Maastricht University, Maastricht, Netherlands; ^6^Adelante, Centre of Expertise in Rehabilitation and Audiology, Hoensbroek, Netherlands; ^7^Medtronic, Maastricht, Netherlands; ^8^University College Leuven Limburg, Diepenbeek, Belgium

**Keywords:** tinnitus perception, tinnitus reactivity, threat, anxiety, negative affect, noise sensitivity, subclinical hearing loss

## Abstract

**Aims and hypotheses:**

In an environment of absolute silence, researchers have found many of their participants to perceive phantom sounds (tinnitus). With this between-subject experiment, we aimed to elaborate on these research findings, and specifically investigated whether–in line with the fear-avoidance model of tinnitus perception and reactivity–fear or level of perceived threat influences the incidence and perceptual qualities of phantom sound percepts in an anechoic room. We investigated the potential role of individual differences in anxiety, negative affect, noise sensitivity and subclinical hearing loss. We hypothesized that participants who experience a higher level of threat would direct their attention more to the auditory system, leading to the perception of tinnitus-like sounds, which would otherwise be subaudible, and that under conditions of increased threat, narrowing of attention would lead to perceptual distortions.

**Methods:**

In total, *N* = 78 normal-hearing volunteers participated in this study. In general, the study sample consisted of young, mostly female, university students. Their hearing was evaluated using gold-standard pure tone audiometry and a speech-in-noise self-test (Digit Triplet Test), which is a sensitive screening test to identify subclinical hearing loss. Prior to a four-minute stay in an anechoic room, we randomized participants block design-wise in a threat (*N* = 37) and no-threat condition (*N* = 41). Participants in the threat condition were deceived about their hearing and were led to believe that staying in the room would potentially harm their hearing temporarily. Participants were asked whether they perceived sounds during their stay in the room and rated the perceptual qualities of sound percepts (loudness and unpleasantness). They were also asked to fill-out standardized questionnaires measuring anxiety (State–Trait Anxiety Inventory), affect (Positive and Negative Affect Schedule) and noise sensitivity (Weinstein Noise Sensitivity Scale). The internal consistency of the questionnaires used was verified in our study sample and ranged between *α* = 0.61 and *α* = 0.90.

**Results:**

In line with incidence rates reported in the literature, 74% of our participants reported having heard tinnitus-like sounds in the anechoic room. Speech-in-noise identification ability was comparable for both groups of participants. The experimental manipulation of threat was proven to be effective, as indicated by significantly higher scores on a Threat Manipulation Checklist among participants in the threat condition as compared to those in the no-threat condition (*p* < 0.01). Nevertheless, participants in the threat condition were as likely to report tinnitus percepts as participants in the no-threat condition (*p* = 1), and tinnitus percepts were not rated as being louder (*p* = 0.76) or more unpleasant (*p* = 0.64) as a function of level of threat. For participants who did experience tinnitus percepts, a higher level of threat was associated with a higher degree of experienced unpleasantness (*p* < 0.01). These associations were absent in those who did not experience tinnitus. Higher negative affect was only slightly associated with higher ratings of tinnitus unpleasantness (*p* < 0.01).

**Conclusion:**

Whereas our threat manipulation was successful in elevating the level of fear, it did not contribute to a higher percentage of participants perceiving tinnitus-like sounds in the threat condition. However, higher levels of perceived threat were related to a higher degree of perceived tinnitus unpleasantness. The findings of our study are drawn from a rather homogenous participant pool in terms of age, gender, and educational background, challenging conclusions that are applicable for the general population. Participants generally obtained normophoric scores on independent variables of interest: they were low anxious, low noise-sensitive, and there was little evidence for the presence of subclinical hearing loss. Possibly, there was insufficient variation in scores to find effects.

## Introduction

Subjective tinnitus, or the perception of sound(s) in the absence of (an) objectively measurable internal or external sound source(s), is estimated to occur in 10–15% of people, globally (Henry et al., 2020). It is believed to result from (patho-)physiological changes in the cochlea or alongside central auditory pathways (bottom-up mechanisms), and often – but certainly not always – coexists with a (measurable) peripheral hearing loss (Knipper et al., 2013; Langguth et al., 2013). Through a link with higher-order top-down mechanisms (probably originating from the limbic system and attentional circuits), a bothersome manifestation of tinnitus can be induced. The tinnitus percept is amplified through increased vigilance, but can be quite different among individuals depending on emotional state and distress levels (for a recent review, see: Knipper et al., 2021).

Many explanatory models have attempted to describe the mechanisms influencing tinnitus perception and reactivity. Although most people adapt to the *humming*, *buzzing*, or *ringing* sound(s) they experience, in the sense that they are not constantly aware of their tinnitus, for some people, chronic tinnitus can become quite an impactful condition, accompanied by emotional distress (Kleinstäuber and Weise, 2021). Key factors that have been associated with tinnitus distress are cognitive misinterpretations, negative emotional reactivity, and attentional processes (Cima et al., 2011). A cognitive-behavioral model for tinnitus, building on the principles of the influential neurophysiological model of Jastreboff (Jastreboff et al., 1996), is the Fear-Avoidance (FA) model. In short, according to the FA model, catastrophizing thoughts about tinnitus and its consequences provoke tinnitus-related fear responses, may increase tinnitus intensity reports and avoidance behavior, ultimately leading to disability and diminished quality of life (Kleinstäuber et al., 2013).

In the early fifties, Heller and Bergman (1953) were the first to observe the perception of tinnitus by self-stated normal hearing adults in an environment of absolute silence, namely in an anechoic room. Ninety-five percent of their participants experienced tinnitus-like sounds, indicating that tinnitus could be a normal experience, and that its perception is masked by everyday ambient noise. Refining methodological drawbacks of that seminal study, several replication studies have found equivalent results, although reported tinnitus incidence numbers have been somewhat lower, ranging from 64 (Tucker et al., 2005) –68% (Knobel and Sanchez, 2008) to 83% (Del Bo et al., 2008). Elaborating on these findings, Knobel and Sanchez (2008) found that auditory attention influenced healthy participants’ tinnitus perceptions in an anechoic chamber. Their participants performed tasks that either drove their attention away from the auditory modality or toward it. In the non-auditory attention conditions, 20–46% of participants perceived tinnitus, compared to 68% in the auditory attention condition.

Fear or perceived threat is known to affect behavior through selective attentional processes; heightened arousal induces and a shift in prioritization to those aspects that are of greatest immediate importance (Baddeley, 1972; Finucane, 2011; van Steenbergen et al., 2011). Trait anxiety reflects a tendency to respond anxiously to a perceived threat and differs from state anxiety which reflects a momentary emotional state with feelings of tension and anxiety (van der Ploeg, 1982). During episodes of heightened fear, perceptual distortions can occur, which are believed to function as an adaptive psychological mechanism: accentuation of threatening features enables more effective behavior (Rachman and Cuk, 1992). Siegel and Stefanucci (2011) studied the influence of negative affect, defined as a trait tendency to experience a broad range of negative feelings, such as worry and anxiety, and poor self-concept (Watson et al., 1988) on auditory perception in healthy participants. They found that, in negative affect state, participants rated a series of neutral tones with varying pitch as being significantly louder compared to participants in a neutral state. However, loudness and annoyance are not necessarily congruent. Some people experience negative emotional reactions to sounds. Noise-sensitive people, for instance, are more attentive to noise and tend to find noise more threatening than less sensitive people. Furthermore, these persons are believed to have a greater general tendency to being annoyed (~negative affect; Luz, 2005).

The current study aimed to replicate and extend the Heller and Bergman experiment and to investigate the role of individual differences in anxiety, negative affect, and noise sensitivity on tinnitus percepts in an anechoic room within a non-clinical population. We experimentally induced tinnitus-related threat using verbal instructions.

Additionally, we aimed to test the potential influence of subtle hearing difficulties, which are difficult to measure using conventional pure tone audiometry, and which could have been overlooked in previous replication studies. Difficulties with understanding speech in noisy environments can be considered an early sign of hearing loss, and can be present even when detection thresholds are well within clinically accepted ranges of normal hearing usually up to 20–25 dB Hearing Level (dB HL). To this end, we used a speech-in-noise test, which is a sensitive measure to objectify early-stage, subtle or subclinical hearing loss, and functional hearing ability in daily communicative environments (Van Eynde et al., 2016). Hearing loss is an important risk factor for developing tinnitus (Nondahl et al., 2011). A recent longitudinal study demonstrated that poor speech recognition ability in noise (and higher levels of anxiety) are associated with higher degrees of tinnitus annoyance in individuals with new-onset tinnitus (Goderie et al., 2022).

Based on the theoretical considerations mentioned above, we hypothesized that:

1. The majority of participants experience tinnitus percepts in an anechoic room.
2. Participants with poorer speech perception in noise report tinnitus more often compared to participants with better speech perception in noise.
3. Higher perceived threat results in more participants reporting tinnitus percepts, and tinnitus percepts being rated as louder and/or more unpleasant as compared to lower perceived threat.
4. Participants with high anxiety, negative affect, and noise sensitivity report tinnitus-like sounds more often, and perceive these sounds as being louder and/or more unpleasant compared to participants with lower negative affect, anxiety, and noise sensitivity.

## Materials and methods

### Participants

Following the favorable advice of the Social and Societal Ethics Committee of KU Leuven, 90 healthy volunteers without hearing problems and/or tinnitus were invited for this study upon informed consent. We asked participants to not consume drugs or alcohol and to avoid noise or music exposure within the 24 h preceding their study session to avoid temporary hearing threshold elevations. We excluded eight participants with hearing thresholds for one or more frequencies exceeding 25 dB HL, and three participants because of procedural errors. One volunteer ceased participation because she felt uncomfortable with the experimental manipulation. In total, 78 participants (63 women, 15 men) completed the study. Their ages ranged between 18 and 52 years, with an average of 23 years. Most participants (92%) were university students.

### Materials and procedures

Study sessions were organized in the laboratory building of the Physics Research Institute (KU Leuven, Department of Acoustics). One session took about 1 h and consisted of hearing tests, the completion of questionnaires, and a 4-min stay in an anechoic chamber during which participants needed to pay attention to sounds. We carefully debriefed participants at the end of their study visit.

#### Hearing tests

##### Pure tone audiometry

First, air conduction hearing thresholds at octave frequencies from 0.25 to 8 kHz were measured according to the 5-up 10-down Hughson-Westlake method (Carhart and Jerger, 1959), using a Madsen Midimate 622 audiometer connected to calibrated TDH-39 headphones. When all thresholds were lower than or equal to 25 dB HL, the participant’s hearing was deemed clinically normal, and participants were eligible for the study. Participants with thresholds >25 dB HL for one or more frequencies were recommended an ENT-visit upon exclusion.

##### Speech perception in noise

Next, the Flemish Digit Triplet Test (Jansen et al., 2013; Van Eynde et al., 2016) was conducted using a 7-inch Google Nexus tablet connected to calibrated DD65 headphones. In this automated speech-in-noise self-test, 27 triplets (random combinations of 3 monosyllabic digits between 1 and 8, uttered by a female talker) are presented per ear (left ear first) against a speech-shaped background noise with a fixed level of 65 dB SPL. The first triplet is presented at a speech-to-noise ratio of 0 dB, indicating that speech and noise are presented equally intense. Next, according to a 1-up 1-down adaptive procedure, the speech level is decreased or increased in case of correct or incorrect triplet identification, respectively. The test results in ear-specific speech reception thresholds (SRT). The SRT is defined as the speech-to-noise ratio required to achieve a probability of 50% for correct triplet identification. The reference-SRT of normal hearing young adults is −11.7 dB (SD = 0.6 dB).

#### Threat manipulation and randomization

The hearing tests were performed to verify bilateral normal hearing, but also played a key role in the experimental manipulation. This study had a between-subject design: using a randomized block design, participants were allocated to either a threat condition (*N* = 37) or a non-threat condition (*N* = 41). In the threat condition, participants were led to believe that, based on the results of the prior hearing tests, that they were marginally eligible to participate and that their stay in the anechoic room could be potentially harmful for their hearing. They were told to have a risk profile and were likely to suffer from temporary side effects, such as temporary high-frequency hearing loss, speech perception difficulties, lowered tolerance for sounds, pain or pressure in the ear, tinnitus, or dizziness. In the non-threat condition, participants were not deceived about their hearing and correctly informed about their results.

#### The anechoic room

Participants were asked to remain seated alone in an anechoic room (123 m^3^) for 4 min, with the lights switched off. This room, with ambient noise levels <15 dB(A) SPL (personal communication), was isolated from external sound sources, with walls absorbing sound to a maximum (> 99% of acoustic energy is absorbed), to simulate an environment of absolute silence. Participants were instructed to pay attention to sounds. Dimming the lights was a methodological choice to encourage participants to focus on auditory sensations and reduce distraction by other stimuli. After 4 min, the lights were turned back on, and the participant was escorted out of the room. In case of extreme discomfort, the participant was able to notify the experimenters to prematurely stop the session.

#### Questionnaires

Before audiometry, we asked participants to fill in two validated questionnaires, namely the Dutch versions of the State–Trait Anxiety Inventory (STAI), measuring state (STAI-S) and trait (STAI-T) anxiety (van der Ploeg, 1982), and the Positive and Negative Affect Schedule (PANAS), measuring positive (PANAS-P) and negative (PANAS-N) affect (Watson et al., 1988). After the anechoic room visit, participants filled in the Weinstein Noise Sensitivity Scale (WNS), measuring noise sensitivity (Weinstein, 1978). Furthermore, two unstandardized questionnaires, developed by Lauren Blockmans and An-Sofie Ceresa (co-authors), were filled in by the participants: one asking for tinnitus perceptions during their stay in the anechoic chamber (Tinnitus Report Questionnaire, TRQ) and one assessing the efficacy of the threat manipulation (Threat Manipulation Checklist, TMC). The TMC an adaptation of a threat manipulation questionnaire used in a previous study (Vlaeyen et al., 2009). Questionnaires were filled out using EMIUM, an online forum developed by the Experimental Psychopathology Research Institute (Janssen, 2008).

##### State–Trait Anxiety Inventory

The STAI is a widely used measure consisting of two times 20 items measuring state and trait anxiety (or anxiety disposition), respectively. Items are rated on a four-point scale. Scores range from 20 to 80, with higher scores being indicative of more severe anxiety. According to the categorization described by Cho et al. (2013), scores of 52 and 54 or less, are considered normal for state and trait anxiety, respectively (Cho et al., 2013). The internal consistency of the STAI in our study sample was verified and deemed acceptable (*α* = 0.69 for state anxiety and *α* = 0.62 for trait anxiety).

##### Positive and negative affect schedule

The PANAS (Watson et al., 1988) is a widely used measure consisting of two 10-item scales measuring positive and negative affect over a certain time frame. Items are rated on a five-point scale. Scores can vary between 10 and 50, with higher scores indicating higher positive or negative affect. Population means for positive and negative affect are 33.3 (*SD* = 7.0) and 17.4 (*SD* = 6.2), respectively. The internal consistency of the PANAS in our study sample was high (*α* = 0.83 for both affective scales).

##### Weinstein noise sensitivity scale

The WNS (Weinstein, 1978) consists of 21 items measuring noise sensitivity. Items are rated on a six-point scale. Scores vary between 21 and 126. Non-noise-sensitive people obtain an average score of 39.8. A high internal consistency (*α* = 0.80) was found for this questionnaire in our study sample.

##### Tinnitus report questionnaire

The TRQ was filled in only by participants who indicated hearing sounds while staying in the anechoic room. It consisted of eight items regarding the number of sounds heard, their subjective description, localization, continuity, loudness (on a scale ranging from 0 to 10, with 10 indicating “*extremely loud*”), and unpleasantness (on a scale ranging from 0 to 10, with 10 indicating “*extremely unpleasant*”).

##### Threat manipulation checklist

To check whether the experimental threat manipulation was effective, participants rated eight statements of thoughts they might have had before entering the anechoic room. More precisely, they were asked to indicate to what extent the statements applied to them on a scale of 0 to 10, with 10 being completely true. Examples of statements are: “*I felt relaxed*”, “*I was wondering whether something bad could happen*”, “*I was afraid of getting dizzy*” etc. Scores on the TMC could vary from 0 to 80. The internal consistency of the manipulation checklist in this study was high (*α* = 0.90).

### Data analysis

**Figure 1:**
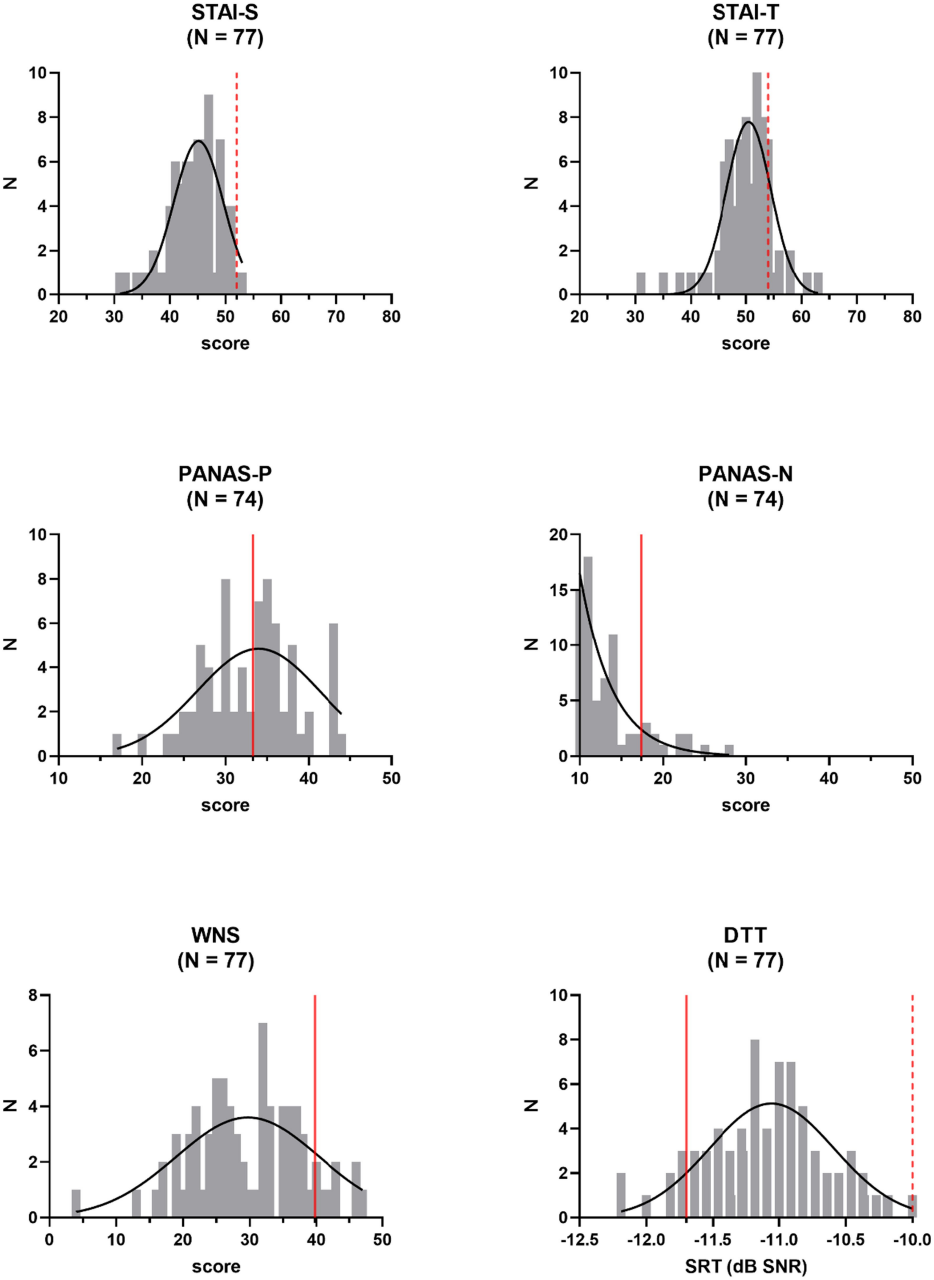
Frequency distributions of independent outcome measures: state (STAI-S) and trait (STAI-T) anxiety, positive (PANAS-P) and negative (PANAS-N) affect, noise sensitivity (WNS), and speech in noise perception (DTT, Digit Triplet Test). Reference values (averages of normative samples) are indicated by the red full lines. Gaussian fits are shown. Pass−/fail-criteria are indicated by the red dashed lines. *N* = number of participants, SRT = speech reception threshold.

Distributions of outcome variables were inspected (Figure 1). We checked whether they met the assumption of normality according to Shapiro–Wilk tests. In case of deviations from a normal distribution (*p* < 0.05), variables were mathematically transformed, and distributions were rechecked. Anxiety and negative affect scores were not normally distributed among study participants for most statistical comparisons, as were TMC-scores and scores for perceived tinnitus loudness or unpleasantness. Taking the square root of the skewed anxiety and negative affect scores did not result in normally distributed variables and did not improve the distribution of the scores for perceptual attributes of perceived tinnitus-like sounds (loudness and unpleasantness). Such a transformation was successful for TMC-scores. Positive affect scores were inverted and added to the scores for items measuring negative affect, to obtain a normally distributed overall measure of negative affect (after square root transformation of the data, with *α* = 0.84). Removing *N* = 2 and *N* = 3 outlying transformed data points (scores deviating >1.5 times the interquartile range from median values) for trait and state anxiety, respectively, resulted in a normal distribution for those measures as well. No outliers were identified in the distribution of scores for tinnitus loudness and unpleasantness to resolve distribution issues. Noise sensitivity scores and speech reception thresholds were normally distributed and were not altered.

Group comparisons, e.g., threat versus non-threat condition participants, or participants with tinnitus perceptions in the anechoic room versus participants without tinnitus perceptions, were performed using one-way Analyses of Covariance (ANCOVA), covarying for gender and age to increase statistical power. A non-parametric Mann-Whitney *U* test was used for the comparison of independent samples when skewedly distributed variables were involved. Non-parametric correlational analyses, using Kendall’s Tau, were conducted, using the raw (untransformed) data, to quantify associations between threat, anxiety, affect, and noise sensitivity, and perceived tinnitus loudness and unpleasantness. Statistical analyses were conducted using the Statistical Package for the Social Sciences (SPSS; IBM, version 27). Graphpad Prism software (version 9) was used for graphical representations.

## Results

### Data description

#### Incidence and perceptual attributes of tinnitus percepts: Qualities, intensity, and unpleasantness

The data of one participant in the non-threat condition, were discarded prior to analyses. This participant showed inconsistent response behavior on the TRQ regarding the presence or absence of tinnitus perceptions in the anechoic room, which was the primary outcome measure of this study. Of the remaining 77 participants, 57 (74%) reported having perceived at least one sound during their stay in the anechoic room. About half of the participants (51%) reported having heard one single sound. The other half heard two (35%) or (more than) three (14%) sounds. Among the most frequently reported sounds were noises (including sizzling sounds) and pure tones (including whistling and buzzing sounds). Knocking, tapping, ringing, and spattering sounds were less frequently reported. Most sounds were reported to be perceived at the level of the ears. Less frequently, sounds were reported to be heard more centrally or from outside. Somewhat more than half the participants perceived tinnitus-like sounds continuously and at constant strength (–60%). For others, the percept was more variable. Most participants (91%) rated the perceived loudness of their tinnitus-like perceptions as being rather soft, i.e., less than 5 on the 0–10 scale. Similarly, 84% of participants rated the experienced unpleasantness as rather low, i.e., less than 5 on the 0–10 scale.

#### Affect, anxiety, noise sensitivity, and speech perception in noise

With average STAI-scores of 44.4 (*SD* = 4.6) and 49.9 (*SD* = 5.0) for state and trait anxiety, respectively, anxiety was low among study participants. Similarly, the average negative affect score of 13.5 (*SD* = 4.1) on the PANAS indicated a rather low degree of negative affect. With a mean value of 33.0 (*SD* = 5.7), positive affect scores were centered around reference values. The average recalculated score for negative affect (PANAS-Nr) was 40.6 (*SD* = 7.8). Participants were also low noise-sensitive, with an average WNS score of 29.7 (*SD* = 8.3). With respect to speech intelligibility in noise, compared to published normative values for young adults, study volunteers performed rather poorly, with a mean SRT-value of −11.1 dB SNR (*SD* = 0.5 dB). However, obtained SRT-values fell well below the advised pass/fail-criterion of −10.0 dB SNR. None of these descriptive measures differed across participants in the threat and the non-threat condition, according to a one-way ANCOVA, controlling for gender and age (STAI-S: Δ = 0.07, *F*(1,70) = 0.96, *p* = 0.33; STAI-T: Δ = 0.09, *F*(1,71) = 1.74, *p* = 0.19; PANAS-Nr: Δ = 0.09 *F*(1,69) = 0.40, *p* = 0.53; WNS: Δ = 2.90, *F*(1,73) = 2.33, *p* = 0.13; DTT-SRT: Δ = 0.06 dB, *F*(1,73) = 0.34, *p* = 0.56). Effect sizes were small with values for partial *η*^2^ ranging from 0.01 to 0.06. Except for PANAS-Nr (*p* < 0.01), the covariate of age was never significant. The gender covariate never reached significance in the above-mentioned analyses.

### Threat

#### Experimental manipulation check

**Figure 2:**
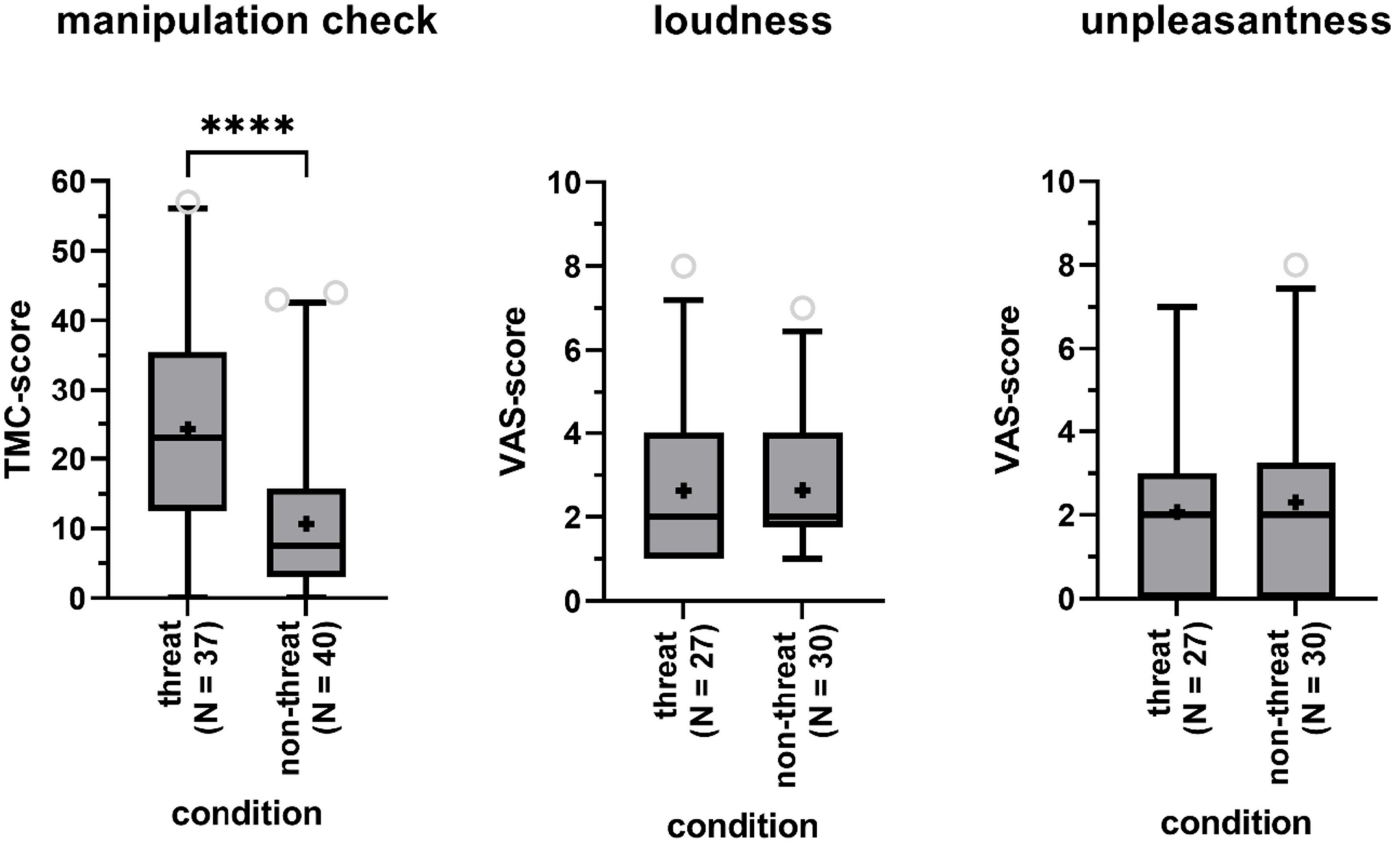
Boxplots of the total score on the Threat Manipulation Checklist (TMC, left panel), the Visual Analog Scales (VAS) for perceived tinnitus loudness (central panel) and unpleasantness (right panel) for participants in the threat and non-threat condition. Boxes include 90% of the data. Outliers are represented by the open gray circles. Median scores are indicated by the black horizontal lines. Crosses represent mean scores. Significant differences are indicated by asterisks (****).

To investigate whether the experimental manipulation of fear was successful, we compared scores on the TMC obtained from participants in the threat condition to those from participants in the non-threat condition. According to a one-way ANCOVA, controlling for gender and age, scores were significantly higher in the threat condition (Δ = 1.85, *F*(1,73) = 22.96, *p* < 0.01) with a large effect size (partial *η*^2^ = 0.24), indicating that participants in that condition experienced a higher degree of threat (Figure 2). Age was a significant covariate in the statistical model (*p* < 0.05), with older participants scoring lower on the TMC.

#### Effect of threat on tinnitus incidence, loudness, and unpleasantness

A Fisher’s Exact Test showed that participants in the threat condition were as likely to report tinnitus-like sounds as participants in the non-threat condition (73% versus 75%, *p* = 1). Among participants reporting to have heard sounds in the anechoic room, sounds were not rated as being louder (mean Δ = 0, *Z* = −0.30, *p* = 0.76) nor being more unpleasant (mean Δ = 0.23, *Z* = −0.48, *p* = 0.64) in the threat versus non-threat condition, according to Mann-Whitney *U*-tests (Figure 2). TMC-scores, indicating perceived level of threat, were not significantly higher for participants with tinnitus perceptions compared to participants without tinnitus perceptions according to a one-way ANCOVA (Δ = 0.90, *F*(1,73) = 3.30, *p* = 0.07, partial *η*^2^ = 0.04), when controlling for age (*p* = 0.05) and gender (*p* = 0.18).

**Figure 3:**
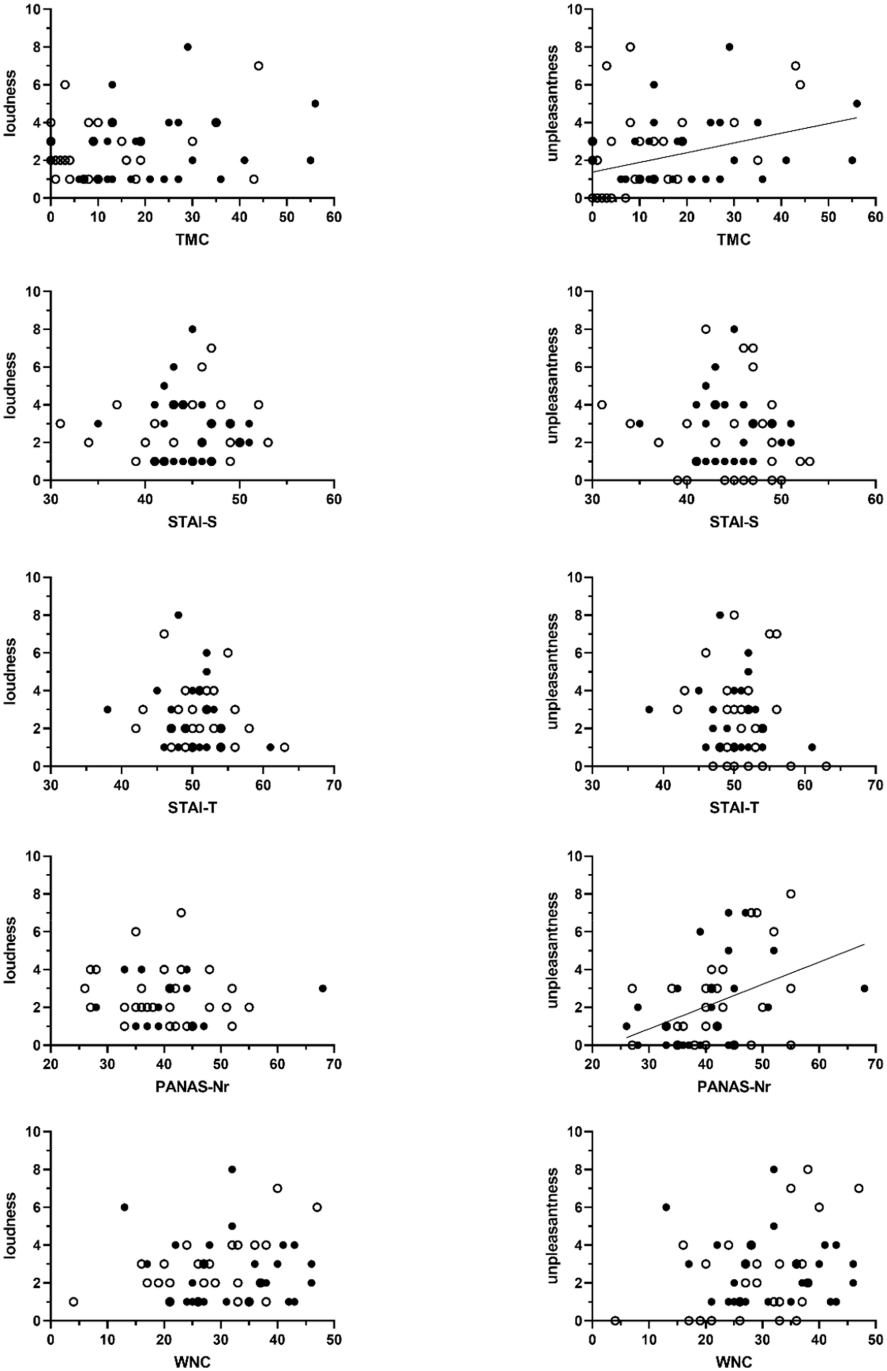
Scatter plots of the association between sum scores on the Threat Manipulation Checklist (TMC), the State–Trait Anxiety Inventory (STAI, STAI-S = state anxiety, STAI-T = trait anxiety), the Positive and Negative Affect Schedule (PANAS – negative affect), and the Weinstein Noise Sensitivity Scale (WNS) and the VAS-scores of perceived tinnitus loudness (left-sided graphs) and unpleasantness (right-sided graphs). Unfilled circles represent scores obtained from participants in the non-threat condition. Filled circles represent data obtained from participants in the threat condition. Significant associations are marked with a regression line. This line shows the association across all participants.

As shown in Figure 3 TMC-scores were significantly associated with tinnitus unpleasantness, with a non-parametric Kendall’s Tau correlation coefficient of 0.30 (*p* < 0.01, *N* = 57). No association with perceived loudness was found (*τ* = 0.10, *p* = 0.32, *N* = 57).

### Individual differences

#### Effects of anxiety, affect, noise sensitivity, and perceived level of threat on tinnitus incidence, loudness, and unpleasantness

**Figure 4:**
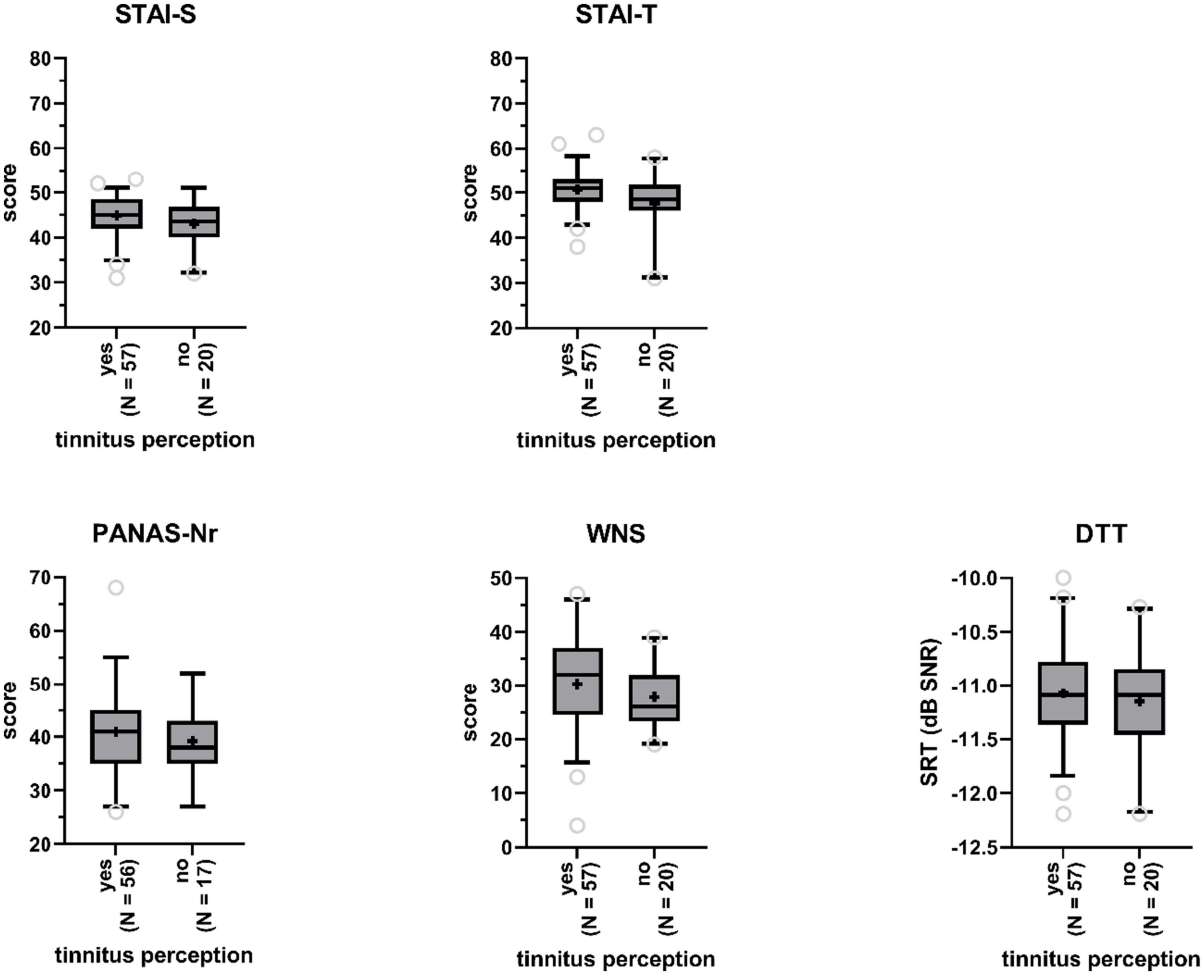
Boxplots of sum scores on the State–Trait Anxiety Inventory (STAI, STAI-S = state anxiety, STAI-T = trait anxiety), the Positive and Negative Affect Schedule (PANAS - negative affect), and the Weinstein Noise Sensitivity Scale (WNS), and Digit Triplet Test (DTT) speech reception thresholds (SRT) for participants reporting tinnitus perceptions and participants reporting no tinnitus perceptions. Boxes include 90% of the data. Outliers are represented by the open gray circles. Median scores are indicated by the black horizontal lines. Crosses represent mean scores.

We investigated whether participants with tinnitus percepts in the anechoic room differed from participants without, with respect to (state and trait) anxiety, negative affect, and noise sensitivity (Figure 4). One-way ANCOVA, controlling for age and gender, indicated that this was not the case: not for state (STAI-S Δ = 0.14 *F*(1,70) = 2.93, *p* = 0.09) nor for trait (STAI-T Δ = 0.10 *F*(1,71) = 1.53, *p* = 0.22) anxiety, negative affect (PANAS-Nr Δ = 0.07, *F*(1,69) = 0.17, *p* = 0.68), and noise sensitivity (WNS Δ = 2.68, *F*(1,73) = 1.50, *p* = 0.23). Effect sizes were small with values for partial *η*^2^ ranging between 0 and 0.04. Age was a highly significant covariate in the model for negative affect (*p* < 0.01), with older participants demonstrating lower negative affect.

Non-parametric correlational analyses did not show significant associations between experienced tinnitus loudness and scores for anxiety (*N* = 57; STAI-S: *τ* = 0.01, *p* = 0.92; STAI-T: *τ* = −0.08, *p* = 0.44), negative affect (*N* = 56; PANAS-Nr: *τ* = 0.15, *p* = 0.14), and noise sensitivity (*N* = 57; WNS: *τ* = 0.10, *p* = 0.17). Significant associations with tinnitus unpleasantness were found. Higher negative affect was associated with tinnitus sounds being perceived more unpleasant (*N* = 56; PANAS-N: *τ* = 0.29, *p* < 0.01). For anxiety (*N* = 57; STAI-S: *τ* = −0.13, *p* = 0.20; STAI-T: *τ* = −0.12, *p* = 0.24) and noise sensitivity (*N* = 57; WNS: *τ* = 0.14, *p* = 0.16) no such associations were found (Figure 3).

#### Effects of subclinical hearing loss on tinnitus incidence

SRTs obtained by participants who reported to have heard tinnitus-like sounds in the anechoic room (*M* = −11.1, *SD* = 0.5) were not significantly poorer than SRTs obtained by participants who did not experience tinnitus in the room (*M* = −11.2, *SD* = 0.5), according to a one-way ANCOVA (*F*(1,73) = 0.37, *p* = 0.55, partial *η*^2^ = 0; Figure 4), when controlling for gender and age.

## Discussion

The primary aim of our study was to replicate and extend the findings of Heller and Bergman (1953), who found that 94% of their non-clinical study population reported to hear tinnitus-like sounds in an anechoic chamber.

Overall, 74% of our participants reported having heard at least one sound while residing 4 min in absolute silence. These results are congruent with the hypothesis that tinnitus is a normal experience when being exposed to an environment with minimal noise levels. This may be due to a change in the functioning and neural organization of central auditory pathways as caused by the temporary sensory deprivation (Salvi et al., 2000; Da Costa, 2001; Knobel and Sanchez, 2008; Schilling et al., 2021). In this light, the perception of tinnitus in a silent environment could be driven by a gain of neuronal activity alongside auditory pathways and reduced background neuronal activity. The reverse can also be true, namely that rather an increase in background activity leads to an increase in information flow along the auditory pathway (Zeng, 2020; Krauss and Tziridis, 2021).

However, and in agreement with other replication studies, our incidence rate of 74% was considerably–and significantly–lower than 94% [95% CI (64.9, 83.1%) (resulting from simple bootstrapping based on *N* = 1,000 samples)]. With reported rates varying between 64 and 83% in replication studies (Tucker et al., 2005; Del Bo et al., 2008; Knobel and Sanchez, 2008), our value lies exactly in the middle of that interval. Compared to the Heller and Bergman study, our participants were younger, and more strictly screened for normal hearing. Next to demographical differences among studies, differences in sample size and time spent in the anechoic room might influence the results. Also, some investigators counted in body sounds (e.g., heartbeat), whereas others did not.

A potential influence of (subclinical) hearing loss can likely be ruled out in this study. Hearing loss, e.g., resulting from noise-induced hearing damage, and tinnitus are auditory complaints that often co-occur. Sometimes, pure-tone thresholds are within normative ranges, while the individual experiences difficulties understanding speech in the presence of background noise. Primary neurodegeneration of the innervated dendrites of the auditory nerve fibers and secondary degeneration of spiral ganglion neurons can be underlying. Subsequent maladaptive neuronal plasticity of the central auditory system can induce tinnitus (Knipper et al., 2013; Moser et al., 2013). With SRTs in noise well below (better than) validated cut-off scores, our participants showed very little evidence for the presence of subclinical hearing loss.

We investigated whether individual differences in anxiety, negative affect and noise sensitivity influence the perception of tinnitus in silence. Our results demonstrate that participants who heard tinnitus-like sounds did not demonstrate higher state anxiety compared to participants who did not report having perceived tinnitus (*p* = 0.09). This is in contrast to findings reported by Cho et al. (2013) who did find a significant association between anxiety and tinnitus distress. Other studies have provided evidence for an association between anxiety sensitivity and tinnitus disability (e.g., Hesser and Andersson, 2009). Anxiety was not associated with the perceived loudness or unpleasantness of experienced tinnitus percepts, but participants with higher negative affect, rated the degree of unpleasantness of their tinnitus percepts as higher. Surprisingly, no similar associations were found with the perceived loudness of tinnitus sounds, as would be expected from the study by Siegel and Stefanucci (2011), who found that persons in a negative affect state rate tones as being significantly louder compared to persons in a neutral state. There is a consensus that, in chronic tinnitus patients, the perceived tinnitus loudness may differ in individuals depending on their emotional state (Knipper et al., 2021). These associations have not yet been explored in non-tinnitus sufferers. Also, methodological differences among studies might introduce mixed findings.

Regarding noise sensitivity, there were no differences among participants with versus without tinnitus percepts in the anechoic room, and there was no association of noise sensitivity scores and perceived tinnitus loudness or unpleasantness. Given that noise-sensitive people attend to and discriminate between sounds more often, an influence of noise sensitivity was expected. Also, noise-sensitive persons react more strongly to sensory stimuli, and adapt to them more slowly (Luz, 2005). Negative affect has been related to these responses: noise-sensitive persons can perceive sounds as being threatening, and have a characteristic tendency of being annoyed, irrespective of exposure to sound (Luz, 2005). Our data do not really back up this notion: noise sensitivity scores and negative affect scores were not associated (*τ* = 0.09, *p* = 0.30), which argues against the negative affect hypothesis of noise sensitivity (Shepherd et al., 2015). Participants with higher negative affect obtained higher scores on the TMC, demonstrating more perceived fear (*τ* = 0.19, *p* = 0.03). However, considering the absolute values of these correlation coefficients, effects sizes are rather small.

Next, to the previously discussed factors, we investigated whether threat would influence tinnitus perception and perceptual attributes (~tinnitus reactions). In their influential cognitive-behavioral model, Mogg and Bradley (1998) state that the threat value of a stimulus is influenced by features of that stimulus, context and the anxiety level of an individual. When perceived as highly threatening, attention will be selectively oriented toward the stimulus. High anxious people allocate a higher subjective threat value to a stimulus, but once evaluated as being threatening, everyone directs attention toward it, regardless of trait anxiety level. Furthermore, under fearful conditions, narrowing of attention can lead to perceptual distortions (Rachman and Cuk, 1992). The principles of the FA model, applied to tinnitus, are in line with this reasoning; a catastrophic interpretation of tinnitus could increase levels of fear and prioritize actions in order to protect against the tinnitus (Cima et al., 2011). In our study, we hypothesized that participants in the threat condition would direct their attention more to the auditory system, leading to perception of tinnitus-like sounds, which would otherwise be subaudible. This hypothesis assumes that participants in the threat condition were under more fearful circumstances than participants in the non-threat condition and were more likely to catastrophize about the perceived sounds. Whereas our threat manipulation was successful in elevating the level of fear, it did not contribute to a higher percentage of participants perceiving tinnitus-like sounds in the threat condition. However, we did find a slight positive association with perceived unpleasantness of tinnitus sounds: higher levels of perceived threat are related to more perceived unpleasantness.

The findings of our study are drawn from a rather homogenous participant pool in terms of age, gender, and educational background (young Caucasian female students), challenging conclusions that are applicable for the general population. Participants generally obtained low scores on independent variables of interest: they were low anxious, low noise-sensitive, and there was little evidence for the presence of subclinical hearing loss. Possibly, there was insufficient variation in scores to find effects on tinnitus perception and perceptual attributes. Study limitations also relate to the experimental manipulation of threat. First, the experiment could have elevated levels of fear in all participants, irrespective of the allocated condition: participants were in an unknown situation, had to enter a dark room where they could ring an alarm when feeling unwell, etc. Second, the manipulation may not have been strong enough to create a threatening condition. Unfortunately, participants did not complete a questionnaire on the credibility of the threat manipulation.

Nonetheless, the findings of this study are novel in the sense that, whereas prior studies have addressed the links between psychological state and tinnitus, none have looked at associations with anxiety and negative affect in normal hearing participants who do not experience tinnitus in daily life, but have it induced by a sound-deprived environment.

Future studies might also want to systematically manipulate the duration of stay in the anechoic room, measure ambient noise levels in the room (and report them), and collect information on daily life noise exposure of study participants (or include participants with different levels of exposure).

## Data availability statement

The raw data supporting the conclusions of this article will be made available by the authors, without undue reservation.

## Ethical statement

The studies involving human participants were reviewed and approved by EC UZ/KU Leuven. The patients/participants provided their written informed consent to participate in this study.

## Author contributions

JV, NV, RC, and TF: study conception and design. LB and AC: data collection. SD, RC, and JV: analysis and interpretation of the results. SD: draft the manuscript and preparation. All authors contributed to the article and approved the submitted version.

## Funding

JV is supported by the Asthenes research program “From Acute Aversive Sensations to Chronic Bodily Symptoms” a long-term structural Methusalem funding (METH/15/011) by the Flemish government, Belgium. NV has a senior clinical investigator fund (FWO Flanders FWO 1804816 N). SD and RC are supported by an H2020 grant (UNITI, grant agreement no. 848261).

## Conflict of interest

TEF since 2019 has been employed by Medtronic, Maastricht in a function/role entirely unrelated to this study. At the time the study was conceptualised, data collected and the preliminary analysis conducted TEF declares there was no commercial or financial relationships that could be construed as a potential conflict of interest.

The remaining authors declare that the research was conducted in the absence of any commercial or financial relationships that could be construed as a potential conflict of interest.

## Publisher’s note

All claims expressed in this article are solely those of the authors and do not necessarily represent those of their affiliated organizations, or those of the publisher, the editors and the reviewers. Any product that may be evaluated in this article, or claim that may be made by its manufacturer, is not guaranteed or endorsed by the publisher.
